# Development of self-compatible Chinese cabbage lines of Chiifu through marker-assisted selection

**DOI:** 10.3389/fpls.2024.1397018

**Published:** 2024-05-30

**Authors:** Lichun Chang, Jianli Liang, Xu Cai, Li Zhang, Yufang Li, Jian Wu, Xiaowu Wang

**Affiliations:** State Key Laboratory of Vegetable Biobreeding, Sino-Dutch Joint Laboratory of Horticultural Genomics, Institute of Vegetables and Flowers, Chinese Academy of Agricultural Sciences, Beijing, China

**Keywords:** Chinese cabbage, self-incompatibility, MLPK, HRM (High-Resolution Melting), MAS (Marker-Assisted Selection)

## Abstract

The continuously refined genome assembly of the Chinese cabbage accession Chiifu is widely recognized as the reference for *Brassica rapa*. However, the high self-incompatibility of Chiifu limits its broader utilization. In this study, we report the development of self-compatible Chiifu lines through a meticulous marker-assisted selection (MAS) strategy, involving the substitution of the Chiifu allele of *MLPK* (*M-locus protein kinase*) with that from the self-compatible Yellow Sarson (YS). A YS-based marker (SC-MLPK) was employed to screen 841 *B. rapa* accessions, confirming that all eight accessions with the *mlpk/mlpk* (*mm*) genotype exhibited self-compatibility. Additionally, we designed 131 High-Resolution Melting (HRM) markers evenly distributed across the *B. rapa* genome as genomic background selection (GBS) markers to facilitate the introgression of self-compatibility from YS into Chiifu along with SC-MLPK. Genome background screening revealed that the BC_3_S_1_ population had a proportion of the recurrent parent genome (PR) ranging from 93.9% to 98.5%. From this population, we identified self-compatible individuals exhibiting a high number of pollen tubes penetrating stigmas (NPT) (>25) and a maximum compatibility index (CI) value of 7.5. Furthermore, we selected two individuals demonstrating significant similarity to Chiifu in both genetic background and morphological appearance, alongside self-compatibility. These selected individuals were self-pollinated to generate two novel lines designated as SC-Chiifu Lines. The development of these self-compatible Chiifu lines, together with the SC-MLPK marker and the set of HRM markers, represents valuable tools for *B. rapa* genetics and breeding.

## Introduction

Chinese cabbage (*Brassica rapa* ssp. *pekinensis*) accession Chiifu exhibits high self-incompatibility. Chiifu is the first *Brassica* accession to be sequenced and assembled as reference genome (Chiifu v1.5) (Wang et al., 2011). Advancements in sequencing technologies have led to the successive development of three updated genome assemblies (Chiifu v2.5, v3.0, and v4.0) (Cai et al., 2017; Zhang et al., 2018, 2023). The Chiifu genome serves as a prominent reference in the study of *Brassica* species, necessitating the mass selfed seeds. Additionally, the construction of a Chiifu mutant library assumes great significance, facilitating investigations into essential agronomic traits within *B. rapa*. However, the labor-intensive process of seed production in the highly self-incompatible Chiifu, reliant upon artificial pollination, poses challenges for its widespread application. In addressing this limitation, the development of a self-compatible Chiifu line with a high homozygosity of the genetic background (HGB) plays a crucial role in promoting genomic and genetic breeding studies of *Brassica* species.

The self-incompatibility (SI) in Chinese cabbage is regulated by a multi-allelic *S* locus, encompassing three genes associated with pollen-pistil recognition: *SRK* (*S-locus receptor kinase*), *SCR/SP11* (*S-locus cysteine-rich protein/S-locus protein 11*), and *SLG* (*S-locus glycoprotein*) (Yamamoto et al., 2023). The genetic set of alleles of these genes at the same *S* locus is designated as the ‘*S* haplotype’ (Nasrallah and Nasrallah, 1993). Incompatible reactions occur when the pistil and pollen share the same *S* haplotype. Additionally, *ARC1* (*arm repeat-containing 1*), *THL1* (*thioredoxin-h-like-1*), *MLPK*, and *FER-Rac/Rop* signaling (*FERONIA receptor kinase homolog* or *Rac/Rop guanosine triphosphatase* signaling), not associated with the *S* locus, are involved in the pathways of the SI response (Stone et al., 1999; Cabrillac et al., 2001; Murase et al., 2004; Zhang et al., 2021).

Yellow Sarson (YS) (*B. rapa* ssp. *tricolaris*) carries a recessive mutation of the modifier (*m*) gene, eliminating the SI response in the stigma (Murase et al., 2004). The *M* gene, responsible for this trait, encodes a membrane-anchored protein kinase (MLPK). A SNP induces a specific amino acid substitution (Gly to Arg) at position 194 (G194R) (Murase et al., 2004). The dysfunctional G194R form of MLPK protein in *mm* plants can be restored through the transient expression of *MLPK*, thereby reinstating the ability of *mm* papilla cells to reject self-pollen (Murase et al., 2004). Two distinct forms of *MLPK*, *MLPK form 1* (*MLPKf1*) and *MLPK form 2* (*MLPKf2*), localize to the papilla cell membrane and directly interact with *SRK* to transduce SI signaling (Kakita et al., 2007). The Chiifu accession carries the *S^60^* haplotype on chromosome A07 (Yamamoto et al., 2023), characterized by *MLPK*-dependent self-incompatibility (Ohata et al., 2023). However, the practical challenge of breeding self-compatible Chinese cabbage lines based on *MLPK* remains unresolved.

The fusion of genomics and breeding has accelerated the development of cultivars with enhanced traits (Dou et al., 2022; Liu et al., 2022; Cai et al., 2023; Li et al., 2023). Utilizing trait-specific markers in conjunction with genomic background analysis has proven to be a potent breeding strategy for overcoming SI. Notably, in *Brassica oleracea*, the construction of a linkage map has facilitated the identification of major quantitative trait loci (QTLs) linked to self-compatibility (SC). Moreover, SC genes have been rapidly introduced by marker-assisted backcrossing (MABC) using self-compatibility-specific markers and 36 genomic background selection (GBS) markers (Xiao et al., 2019). The silencing of *SP11* gene within the *S* locus has led to the development of a self-compatible transgenic line in *B. rapa* (Jung et al., 2012). Leveraging the *S* locus in conjunction with 111 SSR markers distributed evenly on a physical map facilitated MABC, yielding a self-compatible line with a compatibility index (CI) of approximately 1.15 in Chinese cabbage (Zheng et al., 2022). Therefore, it is feasible to identify self-compatible accessions through screening *B. rapa* germplasm and subsequently develop self-compatible Chiifu lines via MABC.

HRM technology, capitalizing on the identification of distinct melting curves of PCR products, has emerged as a high throughput, accurate, and cost-effective tool for detecting genetic variations, including single nucleotide polymorphisms (SNPs), insertions or deletions (INDELs), and methylation (Herrmann et al., 2007; White et al., 2007; Distefano et al., 2012). In our previous study, we developed 148 INDEL markers for HRM genotyping in *B. rapa*, thereby expanding the repertoire of HRM markers in *B. rapa* (Chen et al., 2021). However, these HRM markers were solely designed based on a physical map as GBS markers. This result highlighted the advantage of HRM markers as genome background markers for marker-assisted selection.

This study aims to overcome the SI present in the *B. rapa* reference line Chiifu. We developed a functional-related marker (SC-MLPK) for *MLPK*. The accessions with *mlpk/mlpk* (*mm*) genotype identified by SC-MLPK across *B. rapa* subspecies proved all being self-compatible. Leveraging the constructed physical and genetic maps of *B. rapa*, we developed genetic background HRM markers evenly distributed across the genome. Through the combination of genotyping by the SC-MLPK marker and genetic background analysis for MABC, we successfully developed two self-compatible lines, designated as SC-Chiifu Lines by transferring the *mm* allele into Chiifu. Thus, this study provides valuable insights into the practical application of molecular markers for overcoming the SI in *B. rapa*.

## Materials and methods

### Plant materials

**Figure 1:**
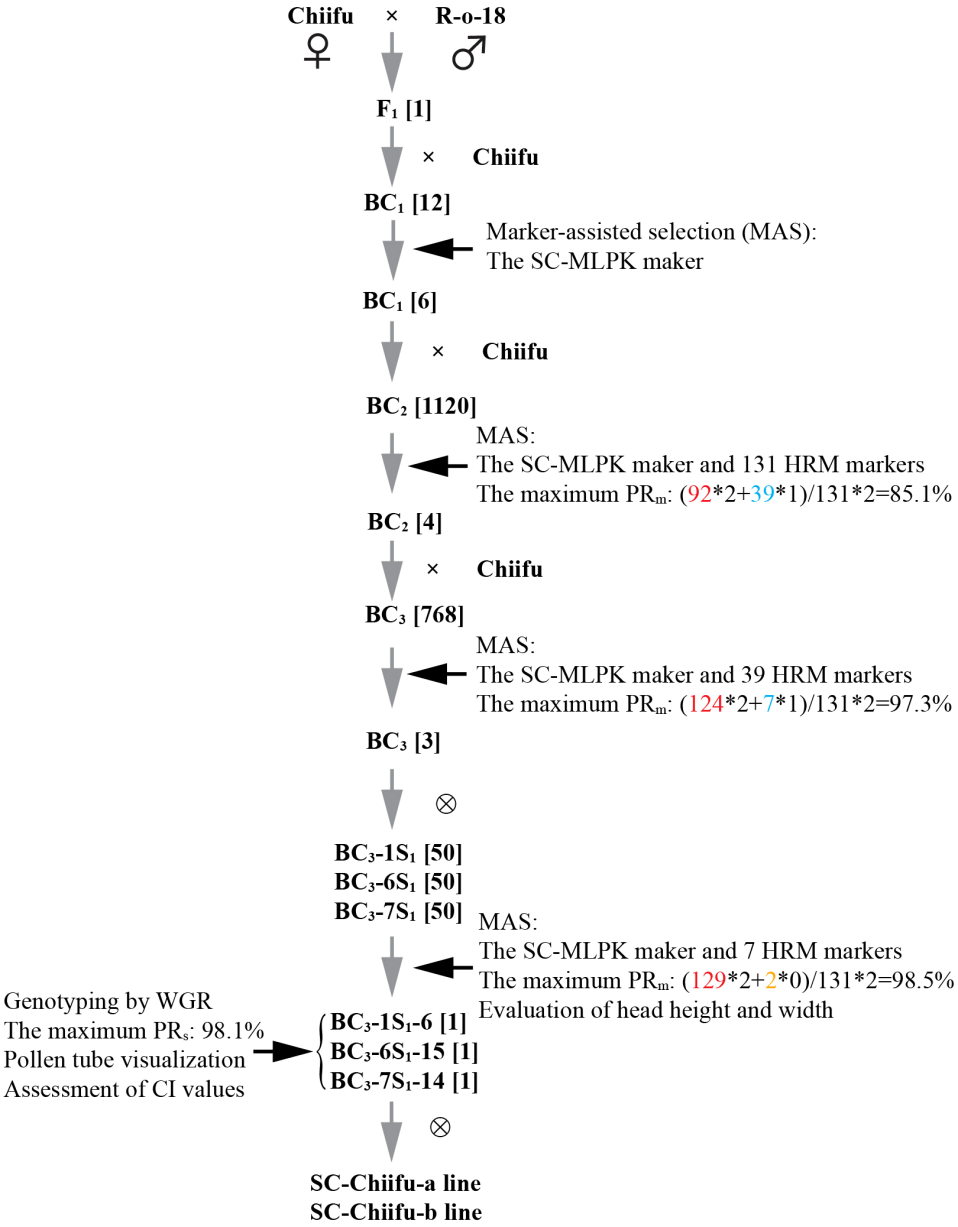
Overcoming high self-incompatibility in Chinese cabbage Chiifu through MABC. The numbers in square brackets indicate the number of plants. Gray arrows represent the process of constructing populations. Black arrows represent the selection by genotyping and phenotyping. The numbers in red, orange and cyan represent the number of markers with Chiifu, R-o-18 or heterozygote genotypes, respectively.

The *B. rapa* reference genome line Chiifu, characterized by a CI value < 1.0 at the anthesis stage, served as the receptor parent. Its *S*-haplotype is *S^60^* (class II), as detailed in a recent study (Yamamoto et al., 2023). A Yellow Sarson line, R-o-18, exhibiting a CI value > 10, was employed as the donor parent. The initial cross involved mating the donor parent R-o-18 with the recurrent parent Chiifu, resulting in the generation of F_1_ plants. Subsequent backcrossing was systematically performed with Chiifu, leading to the development of backcross populations denoted as BC_1_, BC_2_, and BC_3_. From the BC_3_ generation, the BC_3_-1, BC_3_-6, and BC_3_-7 plants with the *Mm* genotype and high proportion of recurrent parent genome (PR) were self-pollinated to yield BC_3_S_1_ populations. Individuals with both phenotypic and genetic background similarity to Chiifu while demonstrating self-compatibility were selected from the BC_3_S_1_ generation to be self-pollinated to produce self-compatible Chiifu lines (**Figure 1**). Additionally, this study included 841 accessions of *B. rapa* from our laboratory to identify individuals with *mm* genotypes. These accessions encompassed 207 Chinese cabbage, 189 Pak choi (ssp. *chinensis*), 150 Caixin (ssp. *parachinensis*), 119 European turnip (ssp. *rapa*), 76 Oil seeds (ssp. *oleifera*), 41 Yellow Sarson (ssp. *tricolaris*), 24 Japanese komatsuna (ssp. *perviridis*), 23 Japanese mizuna (ssp. *nipposinica*), and 12 Taicai (ssp. *chinensis* var. *tai-tsai*) (Cheng et al., 2016).

### DNA extraction and whole-genome resequencing

Genomic DNA was extracted from all samples by the modified CTAB method (Mavrodiev et al., 2021). Plant materials selected for DNA library construction included R-o-18, BC_3_-1S_1_-6, BC_3_-6S_1_-15, and BC_3_-7S_1_-14. These samples were chosen to represent key stages in the breeding program. For WGR, DNA libraries were prepared and subsequently sequenced on a BGISEQ-T7 platform employing paired-end 150 bp (PE150) reads. To ensure the production of high-quality data, the raw sequencing reads were filtered using the fastp software, adhering to default parameters (Chen et al., 2018).

### Variant calls (SNPs/INDELs)

High-quality reads were aligned to the *B. rapa* Chiifu reference genome version 3.0 using Burrows-Wheeler-Alignment (BWA) software with the “mem” method (Li and Durbin, 2009; Zhang et al., 2018). PCR-duplicated reads were filtered out using Samtools software (Li et al., 2009). Variant datasets (SNPs/INDELs) were obtained by applying stringent filters to exclude variants with low quality or multi-allelic features (QUAL < 60, QD < 20, MQ < 30, FS > 13) using GATK software (McKenna et al., 2010; Chang et al., 2023). Additionally, heterozygous loci in the variant datasets of R-o-18 were excluded. Sequence variants of the *MLPK* gene between the reference genome Chiifu v3.0 and R-o-18 aligned reads were visualized using the Integrated Genome Viewer (IGV) software (Robinson et al., 2011). The analysis was conducted with an updated version of genome annotation (Chiifu v3.5) to ensure precision in interpreting the genomic variations (Zhang et al., 2022b).

### *MLPK*-specific marker design and kompetitive allele-specific PCR genotyping

KASP genotyping was employed to discern specific SNPs associated with the *MLPK* gene. Prior to designing the *MLPK*-specific marker, an extensive investigation into SNPs affecting *MLPK* functionality was conducted. Initially, SNPs within *MLPK* were extracted from the SNP dataset of 524 *B. rapa* genomes, as reported in a previous study (Cai et al., 2021). Subsequently, the precise SNP responsible for the G194R mutation within *MLPK*, which has been reported by Murase et al. (2004), was identified. Utilizing this SNP information, an *MLPK*-specific marker was designed using Primer3 software (Untergasser et al., 2012). Furthermore, accessions carrying the *mm* genotype were identified from the SNP dataset of 524 *B. rapa* genomes, with one such accession serving as a positive control for subsequent KASP genotyping.

Amplification and allelic discrimination for the KASP marker were performed using the Scientific QuantStudio™ 12K Flex Real-Time PCR System (Thermo Fisher Scientific, USA). The KASP genotyping protocol followed the methodology outlined in a previous study (Zhang et al., 2022a). Details regarding the KASP reaction volume and thermal cycling conditions for SC-MLPK are provided in **Supplementary Tables S1** and **S2**, respectively.

### Assessment of CI values

The CI value was determined following a previously published protocol (Xiao et al., 2019). Approximately 20 flowers per individual were manually self-pollinated one or two days after reaching full bloom. At the podding stage, CI was calculated using the formula: CI = number of seeds/number of pollinated flowers. Levels of SI, moderate self-compatibility (MSC), or SC were categorized as follows: SI (CI < 1); MSC (1 ≤ CI ≤ 4); SC (CI > 4). *B. rapa* accessions with the *mm* genotype, Chiifu, R-o-18, BC_3_S_1_ individuals with the *mm* genotype, were assessed of CI values. Additionally, from the BC_2_ generation, plants with *Mm* genotypes were self-pollinated to generate BC_2_S_1_ populations. BC_2_S_1_ individuals with the *mm* genotype were assessed of CI values. All pollinations were conducted in the experimental greenhouse of IVF-CAAS, Beijing, China, at around 10:00 a.m. on sunny days.

### Genome-wide HRM marker design and HRM genotyping

Genome-wide INDELs were selected from variant datasets derived between Chiifu and R-o-18. The Primer3 software was employed to design INDEL markers, utilizing 150 bp sequences flanking each INDEL locus (Untergasser et al., 2012; Chen et al., 2021). The designed INDEL markers produced amplicons ranging from 130 bp to 260 bp, with primer lengths between 18 bp and 23 bp. Melting temperature (Tm) predictions for each amplicon were carried out using the nearest-neighbor model (Breslauer et al., 1986; SantaLucia, 1998), setting parameters such as the concentration of sodium ions and reaction temperature to 1 M and 37 °C, respectively. ΔTm for each INDEL amplicon was predicted, and INDEL markers with a ΔTm greater than 0.5°C were retained as HRM markers.

To address the limitations of physical distance in reflecting genetic recombination rates, HRM markers were anchored to the constructed genetic linkage map in *B. rapa* based on their physical positions (Liu et al., 2016). Genome-wide HRM markers, evenly distributed across 10 chromosomes of *B. rapa*, were chosen for HRM genotyping, guided by both physical and genetic maps. Amplification and allelic discrimination for HRM genotyping were performed following a previously reported method (Chen et al., 2021).

### Genetic background analysis using WGR data and HRM markers

WGR data of representative individuals (BC_3_-1S_1_-6, BC_3_-6S_1_-15, BC_3_-7S_1_-14, and R-o-18) were mapped to assembly of Chiifu. Firstly, whole-genome SNP datasets were generated using the Variant Calls method. Loci with missing genotypes were filtered out from the SNP datasets. Genotypes obtained from the SNP datasets were anchored to the physical map based on their respective physical positions. Graphical genotyping analysis was conducted using our Python script available at https://github.com/clc-CAAS/Genetic_map_construction.

PR was calculated after genotyping using the formula: PR = (Recurrent parent allele count * 2 + Heterozygous allele count * 1 + Donor parent allele count * 0)/Total allele count * 2. HGB was calculated after genotyping using the formula: HGB = Homozygous allele count/Total allele count. To distinguish genotyping by HRM markers and WGR data, PR and HGB were designated as PR_m_ and HGB_m_, respectively, when calculated after genotyping by HRM markers, and as PR_s_ and HGB_s_, respectively, when calculated after genotyping by WGR data.

### Pollen tube visualization

Chinese cabbage flowers from Chiifu, R-o-18, BC_3_-1S_1_-6, BC_3_-6S_1_-15, and BC_3_-7S_1_-14 were manually pollinated with a consistent amount of self-pollen grains one or two days after reaching full bloom. Ten flowers were selected for each individual. After 6 hours, stigmas were cut 2 mm away from the stigmatic surface for aniline blue staining to visualize pollen tubes following a published approach (Zhang et al., 2021). Stigmas were fixed in Canoy’s fixative (methanol: acetic acid = 3:1), softened in 10 M NaOH, and stained with 0.1% aniline blue. Pollen tubes were visualized under UV fluorescence using a Leica MSV269 Stereo Microscope, and images were captured with a Leica DFC450 digital camera. SI, MSC or SC was assessed by the number of pollen tubes penetrating stigmas (NPT). The levels of SI, MSC or SC were classified as follows: SI (NPT < 10), MSC (10 ≤ NPT ≤ 25), SC (NPT > 25).

## Results

### Self-compatibility of *mm* plants across 841 *B. rapa* accessions

**Figure 2:**
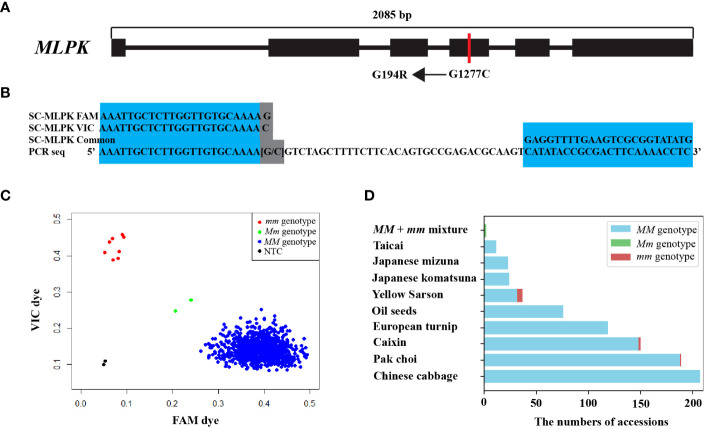
Structural organization of *MLPK* and KASP genotyping by the SC-MLPK marker. **(A)** Structural organization of the *MLPK* gene in *B*. *rapa*, highlighting “G1277C” in the coding sequence (CDS) between Chiifu and R-o-18. “G1277C” represents a single nucleotide substitution (G to C) at position 1277 bp of the *MLPK* gene. “G194R” represents a specific amino acid substitution (Gly to Arg) at position 194. Exons are denoted by black rectangles. **(B)** Sequences of the *MLPK*-specific marker SC-MLPK. **(C)** KASP genotyping in 841 *B*. *rapa* accessions using the SC-MLPK marker. Scattered plots showed clustering of alleles on the X- (FAM) and Y- (VIC) axes. Blue represents FAM-labeled allele (*MM* genotype); red represents VIC labeled allele (*mm* genotype); green represents the *MLPK/mlpk* (*Mm*) genotype, pesudo-heterogenetic sample by manually mixing equal amounts of DNA of R-o-18 and Chiifu; black represents the NTC (no-template control). **(D)** Distribution of *mm* plants across *B*. *rapa* subspecies.

To identify the SNP responsible for inducing G194R within *MLPK*, we examined SNP datasets from 524 *B. rapa* genomes (Cai et al., 2021). We identified a total of 26 SNPs within *MLPK* (**Supplementary Table S3**). A single nucleotide substitution (G to C) at position 1277 bp (G1277C) in the fourth exon of *MLPK* was found to induce the G194R substitution (**Figure 2A**; **Supplementary Table S4**). This G1277C mutation was present in only five out of 524 *B. rapa* genomes, while the *MLPK/MLPK* (*MM*) genotype observed in 509 genomes and missing genotype data in 10 genomes. Additionally, sequence variants of *MLPK* between Chiifu and R-o-18 were visualized, as illustrated in **Supplementary Figure S1**. We developed a specific KASP marker named SC-MLPK based on the G1277C mutation (**Figure 2B**; **Supplementary Table S5**). We optimized the thermal cycling conditions of SC-MLPK for KASP genotyping, determining that 32 cycles after touchdown in the PCR program were superior to the native 26 cycles (**Supplementary Figure S2**; **Supplementary Table S2**).

To identity accessions with *mm* genotypes, we genotyped 841 *B. rapa* accessions by SC-MLPK marker assay. Eight accessions with the *mm* genotype were identified, including five yellow sarson, one pak choi, two caixin. Notably, the *mm* genotype was not detected in any accessions of Chinese cabbage (**Figures 2C, D**). To further confirm the self-compatibility of the *mm* plants, we accessed CI values for these eight accessions (**Table 1**), revealing CI values ranged from 4.25 to 16.3 among them. These results demonstrated that all of the eight accessions with *mm* genotypes were self-compatible. Thus, the *mm* genotype emerged as a potential factor for overcoming self-incompatibility in *B. rapa*.

**Table 1:** The CI value in *mm* plants.

Accessions	Genotype	Type	No. pollinated flowers	No. seed	CI value
611TWXBC	*mm*	Pak choi	20	200	10
6TCX	*mm*	Caixin	20	120	6
T42CX	*mm*	Caixin	20	200	10
Dys 1	*mm*	YS	20	85	4.25
L33	*mm*	YS	20	326	16.3
RARS	*mm*	YS	20	135	6.75
CGN06840	*mm*	YS	20	197	9.85
R-o-18	*mm*	YS	20	230	11.5
Chiifu	*MM*	Chinese cabbage	20	2	0.1

### Development of 131 HRM markers evenly distributed across 10 chromosomes

To facilitate the introgression of *mm* allele into Chiifu through MABC, we developed HRM markers which discriminate genome backgrounds between Chiifu and R-o-18. By aligning 10 Gb WGR data of R-o-18 to the reference genome Chiifu v3.0, a total of 665,095 INDELs were identified. Among these, 188,289 INDELs meeting stringent criteria of homozygosity and a length of ≥ 3 bp were selected for marker design. Furthermore, 131 co-dominant HRM markers (**Supplementary Table S6**) evenly distributed across 10 chromosomes of the *B. rapa* (**Table 2**), were successfully developed for MABC. The ΔTm (melting temperature difference) values of INDEL amplicons between Chiifu and R-o-18 ranged from 0.8 °C to 2 °C for these HRM markers. The number of HRM markers ranged from 10 on chromosomes A07 and A08 to 17 on chromosome A03. The average genome-wide density of HRM markers was 2.28 Mb, ranging from 1.48 Mb on A10 to 3.01 Mb on A09. Furthermore, the average genetic interval across the genome was 7.29 cM, with a range from 5.15 cM on A10 to 9.04 cM on A07 (**Table 2**). The HRM genotyping for a representative HRM marker on each chromosome is depicted in **Supplementary Figure S3**. The 131 evenly distributed HRM markers across 10 chromosomes were used for further MABC of *mm* allele introgression from R-o-18 into Chiifu.

**Table 2:** Total number of HRM markers designed for each *B. rapa* chromosome.

Chromosome	Physical length (bp)	Genetical distance (cM)	No. markers	Average density^1^ (Mb)	Average density^2^ (cM)	Max gap (cM)
A01	29595527	106.84	13	2.28	8.22	13.37
A02	31442979	98.25	12	2.62	8.19	12.31
A03	38154160	111.76	17	2.24	6.57	11.18
A04	21928416	71.58	13	1.69	5.51	8.5
A05	28493056	108.5	14	2.04	7.75	15.62
A06	29167992	98.83	13	2.24	7.6	11.49
A07	28928902	90.36	10	2.89	9.04	13.16
A08	22981702	71.88	10	2.3	7.19	12.43
A09	45156810	115.21	15	3.01	7.68	12.71
A10	20725698	72.11	14	1.48	5.15	9.7

^1^: The average density of HRM markers is calculated based on physical length. ^2^: The average density of HRM markers is calculated based on genetic distance.

### Successful introgression of *mm* allele from R-o-18 into Chiifu

To facilitate the selection of individuals harboring the *Mm* genotype in successive backcrossing populations, the KASP assay for the SC-MLPK marker was applied to the BC_1_, BC_2_, and BC_3_ populations. The distribution of individuals harboring the *Mm* genotype in each backcrossing population was as follows: BC_1_ (6/12), BC_2_ (n=534/1120), and BC_3_ (n=390/768). An extensive population comprising 1120 individuals was utilized in the BC_2_ population to expedite the selection process for plants with the *Mm* genotype and a high PR_m_. The segregation ratio of *MM*: *Mm* in each backcrossing population aligned with the anticipated Mendelian ratio of 1: 1 (*P* > 0.05).

To streamline the genetic background analysis in the extensive BC_2_ population, a two-step MAS strategy was implemented for individuals with the *Mm* genotype. In the first step, three HRM markers on chromosomes A01, A02, and A09 were used for preliminary selection (**Supplementary Figures S4A-D**). Subsequently, a total of 96 individuals selected from the first round screening were upon the second round selection using the left 128 HRM markers. Based on the result from assays by SC-MLPK and 131 HRM markers, the maximum PR_m_ in the BC_2_ population was determined to be 85.1%. For background analysis of individuals with the *Mm* genotype in the BC_3_ population, 39 markers with heterozygous genotypes from the BC_2_ population were employed. The highest PR_m_ was 97.3% among the BC_3_ population (**Supplementary Figure S4D**). These results elucidated that the genetic background of each backcrossing population progressively approached to that of Chiifu.

**Figure 3:**
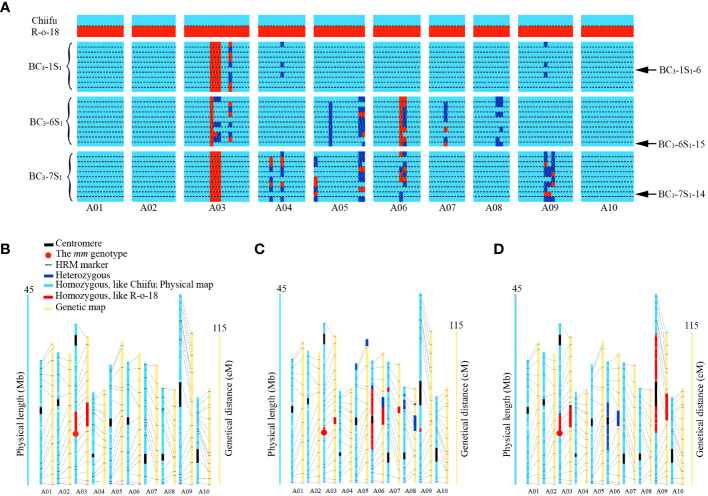
Genetic background analysis using markers and WGR. **(A)** Graphical genotyping based on polymorphism for SC-MLPK and 131 HRM markers. The vertical axis represents parental materials and ten introgression individuals of each BC_3_S_1_ population, while the horizontal axis represents SC-MLPK and 131 HRM markers (totaling 132 markers). Introgression individuals are grouped by different BC_3_S_1_ populations. The markers were organized according to their physical positions on the chromosomes. Arrow represents the selected individuals from each BC_3_S_1_ population. **(B-D)** Genotypes, determined by WGR, anchored to physical and genetic maps in BC_3_-1S_1_-6, BC_3_-6S_1_-15 and BC_3_-7S_1_-14, respectively. The yellow columns represent the genetic linkage groups corresponding to each chromosome. The centromere region is delineated by the black box. The red dot represents the *mm* genotype. Light blue segment indicated the homozygous genotype identical to Chiifu, red segment denoted the homozygous genotype identical to R-o-18, and navy blue segment represented the heterozygous genotype.

Three BC_3_ individuals with *Mm* genotypes as well as high PR_m_, including BC_3_-1 (PR_m_ = 97.3%), BC_3_-6 (PR_m_ = 95.1%), and BC_3_-7 (PR_m_ = 94.7%), were retained for self-pollination to generate BC_3_S_1_ populations. From each BC_3_S_1_ population, ten *mm* genotype plants were selected to be genotyped using background HRM markers (**Figure 3A**). Genotyping revealed that PR_m_ for the BC_3_S_1_ population ranged from 93.9% to 98.5%. Three *mm* individuals with high PR_m_ and high HGB_m_, namely BC_3_-1S_1_-6, BC_3_-6S_1_-15, and BC_3_-7S_1_-14, were retained from BC_3_S_1_ population. Genotyping indicated that the PR_m_ of BC_3_-1S_1_-6, BC_3_-6S_1_-15, and BC_3_-7S_1_-14 was 98.5%, 96.6%, and 95.8%, respectively. The HGB_m_ of BC_3_-1S_1_-6, BC_3_-6S_1_-15, and BC_3_-7S_1_-14 was 100%, 96.2%, and 99.2%, respectively.

To further confirm the high similarity of genetic backgrounds between the three BC_3_S_1_ individuals to that of Chiifu, we conducted WGR for them. Genome-wide SNPs were extracted from aligned reads, utilizing the *B. rapa* genome Chiifu v3.0 as a reference. Loci with missing genotypes for each sample were filtered, and SNPs with homozygous genotypes in R-o-18 were retained, resulting in the identification of 1.6 million SNPs spanning chromosomes A01 to A10. These SNPs were anchored to both physical and genetic maps (**Figures 3B-D**). Based on the SNPs, boundaries of introgressed segments were delineated in BC_3_-1S_1_-6, BC_3_-6S_1_-15, and BC_3_-7S_1_-14 (**Supplementary Table S7**). Genotyping revealed that the PR_s_ of BC_3_-1S_1_-6, BC_3_-6S_1_-15, and BC_3_-7S_1_-14 was 98.1%, 92.6%, and 88.5%, respectively. The HGB_s_ of BC_3_-1S_1_-6, BC_3_-6S_1_-15, and BC_3_-7S_1_-14 was 99.6%, 97%, and 96%, respectively (**Table 3**). The individual plant (BC_3_-1S_1_-6) with the highest PR_s_ of 98.1% in the BC_3_S_1_ population displayed a near-identical match with the recurrent parent Chiifu genotype, except for a specific region (approximately 5.2 Mb, from 12059865 to 17244940) of the *MLPK* gene linkage on chromosome A03 retaining the donor parent R-o-18 genotype (**Figure 3B**). This result indicated successful introgression of *mm* allele from R-o-18 into Chiifu.

**Table 3:** PR and HGB in three selected individuals.

Individual	Genotype	PR_m_ (%)	HGB_m_ (%)	PR_s_ (%)	HGB_s_ (%)
BC_3_-1S_1_-6	*mm*	98.5	100	98.1	99.6
BC_3_-6S_1_-15	*mm*	96.6	96.2	92.6	97
BC_3_-7S_1_-14	*mm*	95.8	99.2	88.5	96
R-o-18	*mm*	0	100	0	100
Chiifu	*MM*	100	100	100	100

### Novel self-compatible Chiifu lines

**Figure 4:**
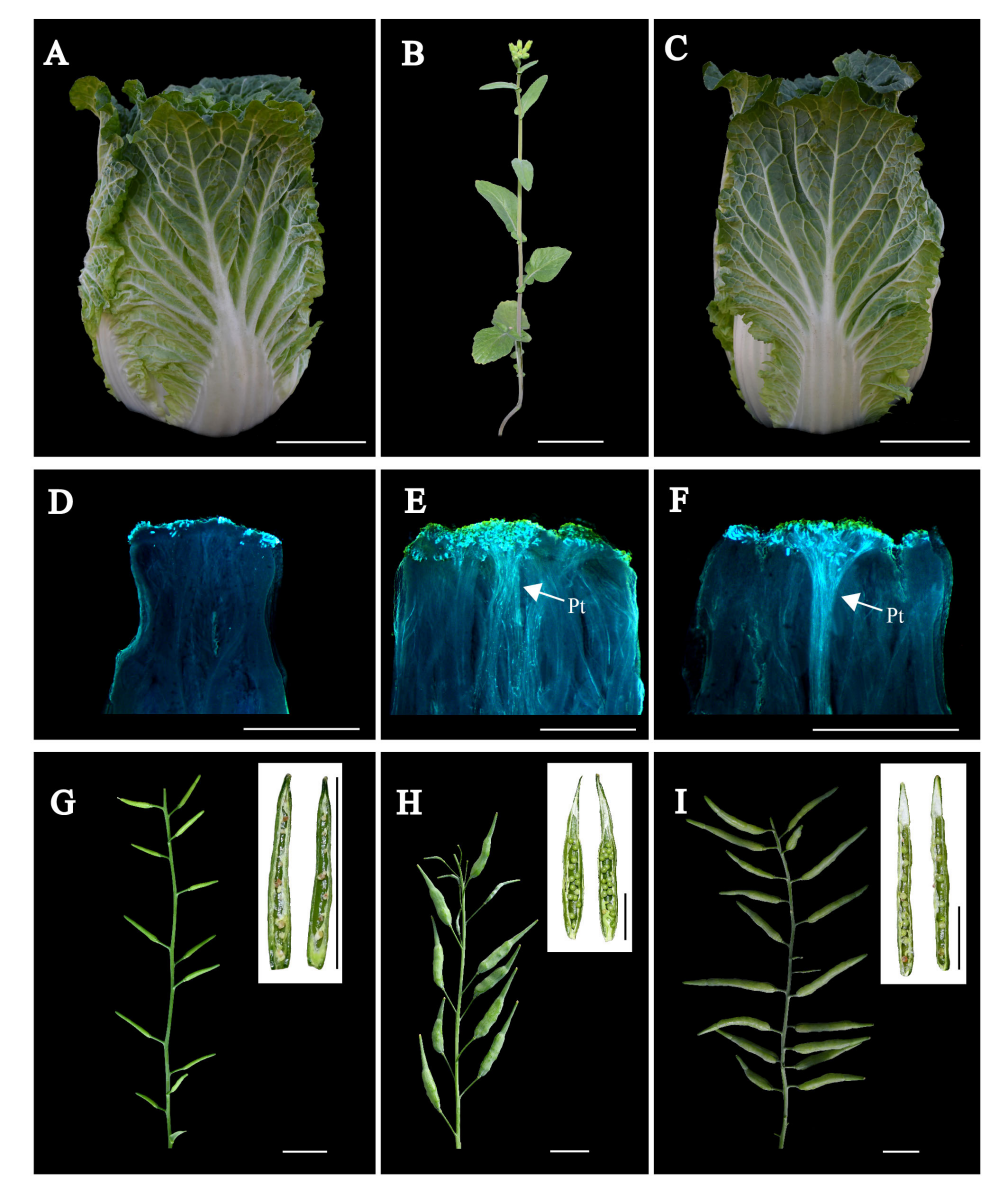
Comprehensive assessment of field and reproductive performance in Chiifu, R-o-18 and BC_3_-6S_1_-15 individual plants. **(A–C)** Field performance comparison of Chiifu, R-o-18, and BC_3_-6S_1_-15 plants. Scale bars in **(A–C)**, 5 cm. **(D–F)** Fluorescence microscopic images depicting pollen tube germination at the stigmas of Chiifu, R-o-18, and BC_3_-6S_1_-15 individual plants, with “Pt” indicating the pollen tubes. Scale bars in **(D–F)**, 1 mm. **(G-I)** Seed setting of Chiifu, R-o-18, and BC_3_-6S_1_-15 individual plants. Scale bars in **(G–I)**, 2 cm.

To evaluate the phenotypic traits of individual plants within the BC_3_-1S_1_, BC_3_-6S_1_, and BC_3_-7S_1_ populations relative to Chiifu, we conducted a detailed analysis encompassing agronomic traits, pollen tube visualization, and assessment of CI value (**Figure 4**). For agronomic trait analysis, 10 plants with *mm* genotype were selected from each BC_3_S_1_ population. Before leafy heads were ready for harvest, head height and width were evaluated and compared with Chiifu (**Table 4**). The head height and width ranged from 23.5 to 25.1 cm and 10.7 to 13.0 cm, respectively. Notably, both the average head height and width in BC_3_-6S_1_ population were not significantly different from those of Chiifu. In each BC_3_S_1_ population, all individuals exhibited heading, hairy, and crinkle traits similar to Chiifu, while the R-o-18 line displayed non-heading, hairless, and smooth traits. Three individuals including BC_3_-6S_1_-15, BC_3_-1S_1_-6, and BC_3_-7S_1_-14 exhibiting both genotypic and phenotypic similarity to Chiifu were selected from each BC_3_S_1_ population (**Figures 4A-C**; **Supplementary Figure S5**).

**Table 4:** Analysis of agronomic traits in three BC_3_S_1_ populations.

	Line/population (Average ± SD)				
	BC_3_-1S_1_ (n=10)	BC_3_-6S_1_ (n=10)	BC_3_-7S_1_ (n=10)	Chiifu (n=10)	R-o-18 (n=10)
Head height (cm)	24.20 ± 1.64 ns	23.50 ± 1.36 ns	25.10 ± 0.79 *	23.70 ± 1.37	–
Head width (cm)	10.65 ± 1.13 *	11.35 ± 1.06 ns	13.00 ± 1.21 *	11.75 ± 0.49	–
Leaf head	Heading (100%)	Heading (100%)	Heading (100%)	Heading (100%)	Non-heading (100%)
Leaf hair	Hairy (100%)	Hairy (100%)	Hairy (100%)	Hairy (100%)	Hairless (100%)
Leaf crinkle	Crinkle (100%)	Crinkle (100%)	Crinkle (100%)	Crinkle (100%)	Smooth (100%)

A two-tailed t test was used to calculate significance between Chiifu line and each BC_3_S_1_ population. ns not significant; *p<0.05.

Upon flowering, BC_3_-6S_1_-15, BC_3_-1S_1_-6, and BC_3_-7S_1_-14, together with Chiifu, R-o-18, were investigated for pollen tube elongation and CI. Fluorescence microscopy revealed that R-o-18, BC_3_-6S_1_-15, and BC_3_-7S_1_-14 plants had more than 25 pollen tubes, whereas no pollen tubes were observed in BC_3_-1S_1_-6 and Chiifu (**Figures 4D-F**; **Supplementary Figure S6**). During the seed-filling stage, seed setting was evaluated (**Figures 4G-I**). Notably, BC_3-_6S_1_-15 and BC_3_-7S_1_-14 plants exhibited significant SC, achieving CI values of 7.5 and 7.1, respectively. In contrast, BC_3_-1S_1_-6 exhibited SI with a CI of 0.1, similar to that of Chiifu (CI = 0.1). Consequently, the two newly developed plants (BC_3_-6S_1_-15, BC_3_-7S_1_-14), which exhibited phenotypic and genetic similarities to Chiifu while demonstrating SC, were self-pollinated to generate two novel lines designated as SC-Chiifu Lines (SC-Chiifu-a, SC-Chiifu-b, respectively). The CI values of the SC-Chiifu Lines compared to the CI value of Chiifu (CI = 0.1) increased tenfold. However, the CI values of the SC-Chiifu Lines remained lower than that observed for R-o-18 (CI = 12). These results highlight the successful transfer of self-compatibility from R-o-18 into Chiifu, leading to the development of two novel SC-Chiifu Lines.

### Segregation of SC in *mm* plants

In the BC_2_S_1_ population, 63 individuals with the *mm* genotype were selected for the assessment of CI values. The CI values varied significantly among these individuals, spanning from 0.2 to 13 (**Supplementary Table S8**). Subsequent CI analysis delineated 29 individuals exhibiting SC (CI > 4), 22 individuals displaying MSC (1 ≤ CI ≤ 4), and 12 individuals manifesting SI (CI < 1). This observation indicated a pronounced segregation of SC within the *mm* plants of the BC_2_S_1_ population.

**Figure 5:**
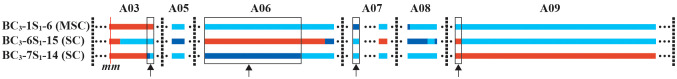
Identification of the shared segments in BC_3_-6S_1_-15 and BC_3_-7S_1_-14 but not in BC_3_-1S_1_-6. Light blue segments indicated the homozygous genotype identical to Chiifu; navy blue segments indicate the heterozygous genotype; red segments indicate the homozygous R-o-18 genotype. Arrows indicate the shared segments between BC_3_-6S_1_-15 and BC_3_-7S_1_-14.

Furthermore, variations in SC were also observed among the three selected individuals (BC_3_-6S_1_-15, BC_3_-7S_1_-14, and BC_3_-1S_1_-6) with the *mm* genotype. Given that all three plants in the BC_3_S_1_ population possessed the same *mm* genotype and similar genetic backgrounds, the observed segregation suggested the involvement of gene(s) beyond the *MLPK* locus in influencing self-compatibility in *B. rapa*. We postulated that the gene(s) responsible being located at the segments shared by BC_3_-6S_1_-15 and BC_3_-7S_1_-14 individuals are distinct from those in BC_3_-1S_1_-6. We examined *S* haplotypes in BC_3_-1S_1_-6, BC_3_-6S_1_-15, BC_3_-7S_1_-14, Chiifu and R-o-18 using *S-*specific primers (Oikawa et al., 2011). We found that BC_3_-1S_1_-6, BC_3_-6S_1_-15, and BC_3_-7S_1_-14, along with Chiifu, had *S^60^* homozygotes, while R-o-18 exhibited an *S^54^* homozygote. There was no correlation between CI value and *S* haplotype in BC_3_-1S_1_-6, BC_3_-6S_1_-15 and BC_3_-7S_1_-14. Based on our analysis on the introgressed segment boundaries in the three selected individuals, we identified four such segments (**Figure 5**). Specifically, four shared segments were located on chromosomes A03 (approximately 0.8 Mb, from 16451403 to 17244940), A06 (approximately 11.3 Mb, from 8101576 to 19449508), A07 (approximately 0.8 Mb, from 14002870 to 14798378), and A09 (approximately 0.7 Mb, from 12255709 to 12997755). These findings suggested that these shared segments emerged as candidate segments potentially influencing SC.

## Discussion

The production of self-pollinated seeds is difficult for Chiifu due to its high self-incompatibility, hampering it being widely used in genetic studies although the reference genome assemblies have been continuously updated. The small bud size of Chiifu adds to the complexity, making it challenging to manually open for self-pollination attempts. Despite efforts to self-pollinate during the bud stage, success rates are often low due to its small bud size and high self-incompatibility. Furthermore, the combination of high self-incompatibility and strong crossing compatibility exacerbates the issue by frequently leading to presence of contaminated hybrid seeds in the limited seed yield obtained from self-pollination attempts. In this study, we addressed the self-incompatibility issue in Chiifu by introducing the *mm* allele into this line. Through this genetic modification, we successfully developed two SC-Chiifu lines that not only retained genetic backgrounds closely resembling Chiifu but also exhibited self-compatibility phenotypes. The development of these SC-Chiifu lines enabled efficient seed setting without the need for manual pollination. This not only alleviates the challenges associated with self-pollination but also mitigates the risk of cross-pollen contamination.

Genetic background analysis within the SC-Chiifu Lines revealed that approximately 90% of the genetic segments originate from the recurrent parent, Chiifu, with the remaining 10% comprising exogenetic segments from the donor parent, R-o-18. Through the utilization of WGR data, we precisely delineated the boundaries of these exogenetic segments. This delineation offers valuable insights for future research endeavors involving the SC-Chiifu Lines. If the SC-Chiifu Lines are employed for updating the Chiifu genome, it is imperative to purify these exogenetic segments through targeted selection. Alternatively, for functional gene research, it may be feasible to disregard these exogenetic segments if they do not significantly impact the traits of interest, such as heading and leaf development. The SC-Chiifu Lines represent crucial plant materials for various applications, including the construction of a Chiifu mutant library and conducting Chiifu-based transgenic experiments. These lines are particularly valuable for breeding self-compatible heading Chinese cabbage, serving as ideal donor parents to provide self-compatibility and heading traits. In the study of self-compatibility mechanisms, both self-compatible SC-Chiifu lines and the self-incompatible BC_3_-1S_1_-6 individual have proven to be valuable resources. Furthermore, the seeds from the SC-Chiifu Lines described in this study will be made accessible to researchers for further functional genomics studies, thereby facilitating broader exploration and utilization of these valuable genetic resources.

*B. rapa* typically exhibits SI, overcoming this trait is not only crucial for utilizing the Chiifu line but also plays a pivotal role in enhancing *B. rapa* hybrid seed production using self-compatible lines within the male-sterile system. The conversion of male parental lines and maintainer lines into self-compatible lines is essential for the hybrid seed production process. Self-compatible lines streamline the reproduction process through honeybee pollination and circumvent crossing incompatibility during hybrid seed production. The G1277C mutation within *MLPK* represents a rare mutation. Among 841 accessions of *B. rapa* analyzed, only a minority, specifically eight accessions, exhibited the G1277C mutation. Assessment of CI values revealed that these eight accessions all demonstrated SC. In response to the G1277C, we developed a self-compatibility-specific marker, SC-MLPK. Following experimental optimization, this marker exhibited stable and accurate genotyping characteristics, making it widely applicable for the creation of self-compatible *B. rapa* lines. The successful utilization of SC-MLPK for overcoming SI serves as a valuable reference for the cultivation of self-compatible *B. rapa* lines.

The success of MABC critically depends on the development of molecular markers that are not only evenly distributed, operationally straightforward, and cost-effective but also high in density for precise genetic background analysis. HRM genotyping technology offers distinct advantages in meeting these criteria. In a previous study, we developed 148 HRM markers evenly distributed in the genome based on a physical map to differentiate between four heading and four non-heading *B. rapa* (Chen et al., 2021). In this study, we expanded upon this approach by developing 131 HRM markers evenly distributed across the *B. rapa* genome, guided by both physical and genetic maps, to differentiate between Chinese cabbage line Chiifu and yellow sarson line R-o-18. These 279 HRM markers represent a powerful tool in *B. rapa* genetics and breeding.

Notably we observed segregation in SI and SC among plants with *mm* genotypes in the both BC_2_S_1_ and BC_3_S_1_ generations. This is different from the observation in germplasm screening that all the eight accessions with G1277C mutation were SC. These observations suggested the potential for overcoming SI barriers or reinforcing SC through the incorporation of gene(s) beyond *MLPK*. Put differently, under a genetic background akin to Chiifu, individuals carrying solely *mm* genotypes prove inadequate in overcoming its self-incompatibility. To obtain self-compatible Chiifu lines, it is imperative to consider not only the *MLPK* locus but also other loci influencing self-compatibility. This necessity compromises our ability to achieve a PR of 100% in obtaining the SC-Chiifu Lines. Through the identification of boundaries of the three selected individuals in the BC_3_S_1_ generation, we detected four candidate segments potentially regulating SI. Additionally, lines SC-Chiifu-a and SC-Chiifu-b (CI > 7) overcame SI and reached SC levels, albeit not reaching the SC level of R-o-18 (CI = 12). This suggested that besides the gene(s) in the four candidate segments, *S* haplotype and *MLPK*, other gene(s) may influence SC. Further exploration through the construction of genetic populations can elucidate the genetic mechanisms involved in overcoming SI barriers or enhancing SC. Such investigations hold promise for advancing our understanding of SC mechanisms in *Brassica* and for developing more efficient breeding strategies.

## Data availability statement

The datasets presented in this study can be found in online repositories. The names of the repository/repositories and accession number(s) can be found in the article/**Supplementary Material**.

## Author contributions

LC: Data curation, Formal analysis, Investigation, Methodology, Resources, Software, Writing – original draft, Writing – review & editing. JL: Resources, Writing – review & editing. XC: Formal analysis, Writing – review & editing. LZ: Resources, Writing – review & editing. YL: Methodology, Writing – review & editing. JW: Funding acquisition, Project administration, Writing – review & editing. XW: Funding acquisition, Project administration, Resources, Supervision, Writing – review & editing.
