# Supplementary material for: Development of self-compatible Chinese cabbage lines of Chiifu through marker-assisted selection
Source: Front Plant Sci. 2024 May 30;15:1397018. doi: 10.3389/fpls.2024.1397018 (PMC11169807; doi:10.3389/fpls.2024.1397018)
Supplement: Supplementary file 1 [file DataSheet_1.docx]

Supplementary Material

## Supplementary Figures


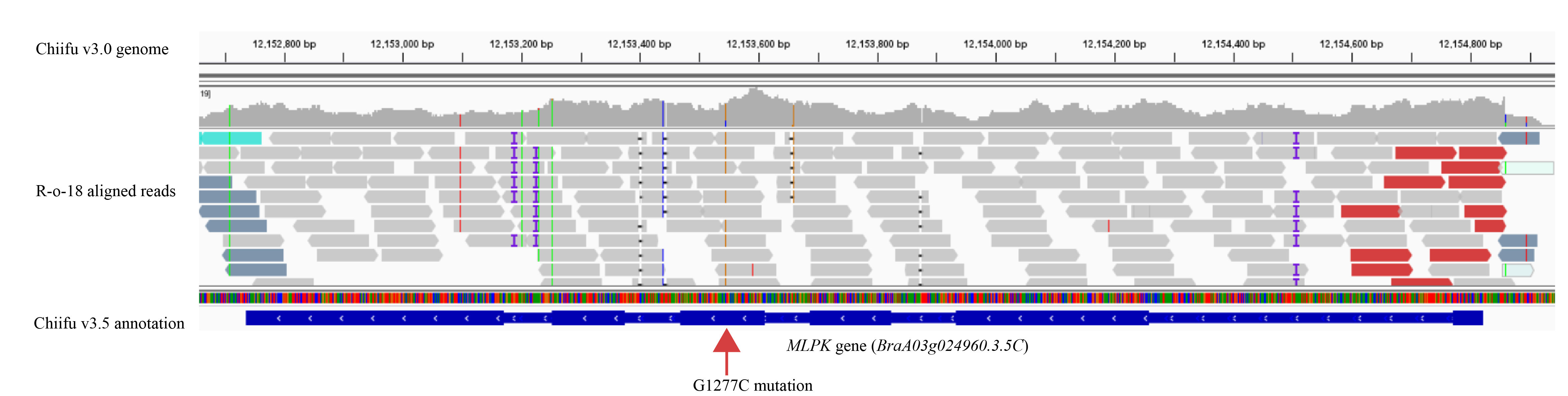


**Supplementary Figure 1.** The G1277C mutation (red arrow) of the *MLPK* gene between Chiifu v3.0 genome and R-o-18 aligned reads.


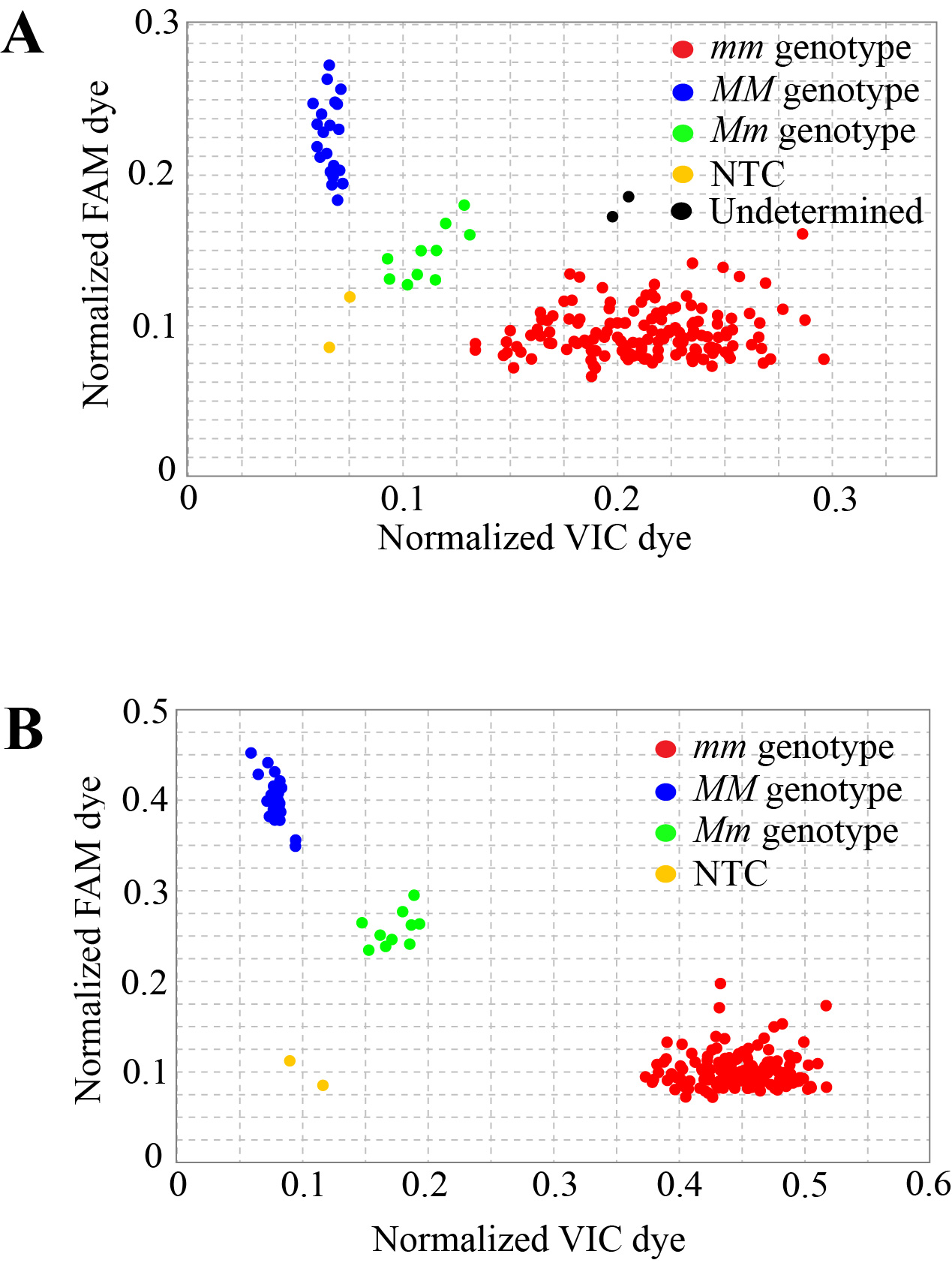


**Supplementary Figure 2.** Optimization of the KASP marker SC-MLPK. **(A)** 26cycles **(B)** 32cycles.


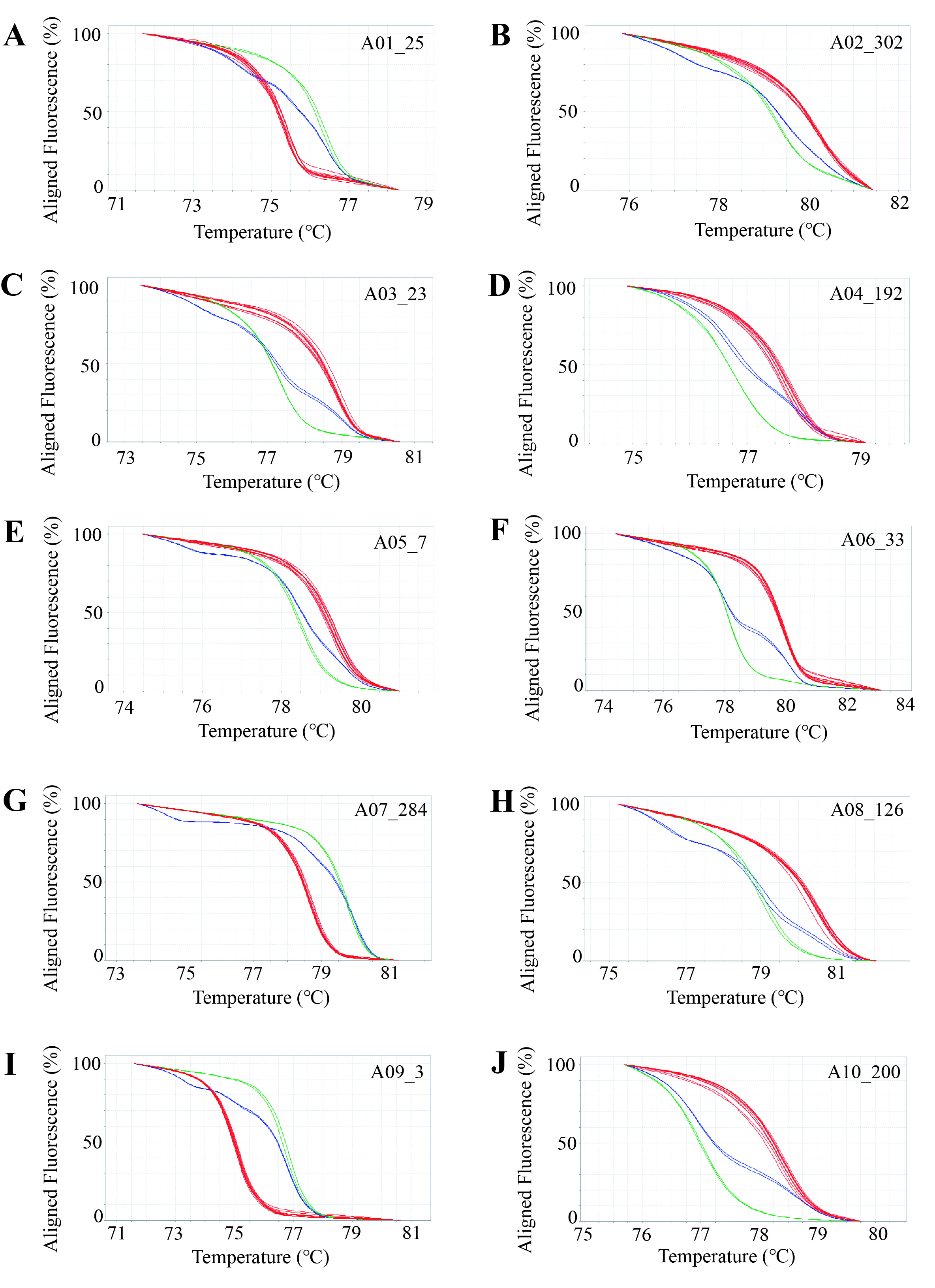


**Supplementary Figure 3.** **(A-J)** Ten polymorphic HRM markers (A01_25, A02_302, A03_23, A04_192, A05_7, A06_33, A07_284, A08_126, A09_3, A10_200) located on ten chromosome. One representative HRM marker on each chromosome. Red curved lines represent the genotypes same as Chiifu. Green curved lines represent the genotypes same as R-o-18. Blue curved lines represent heterogynous genotypes.


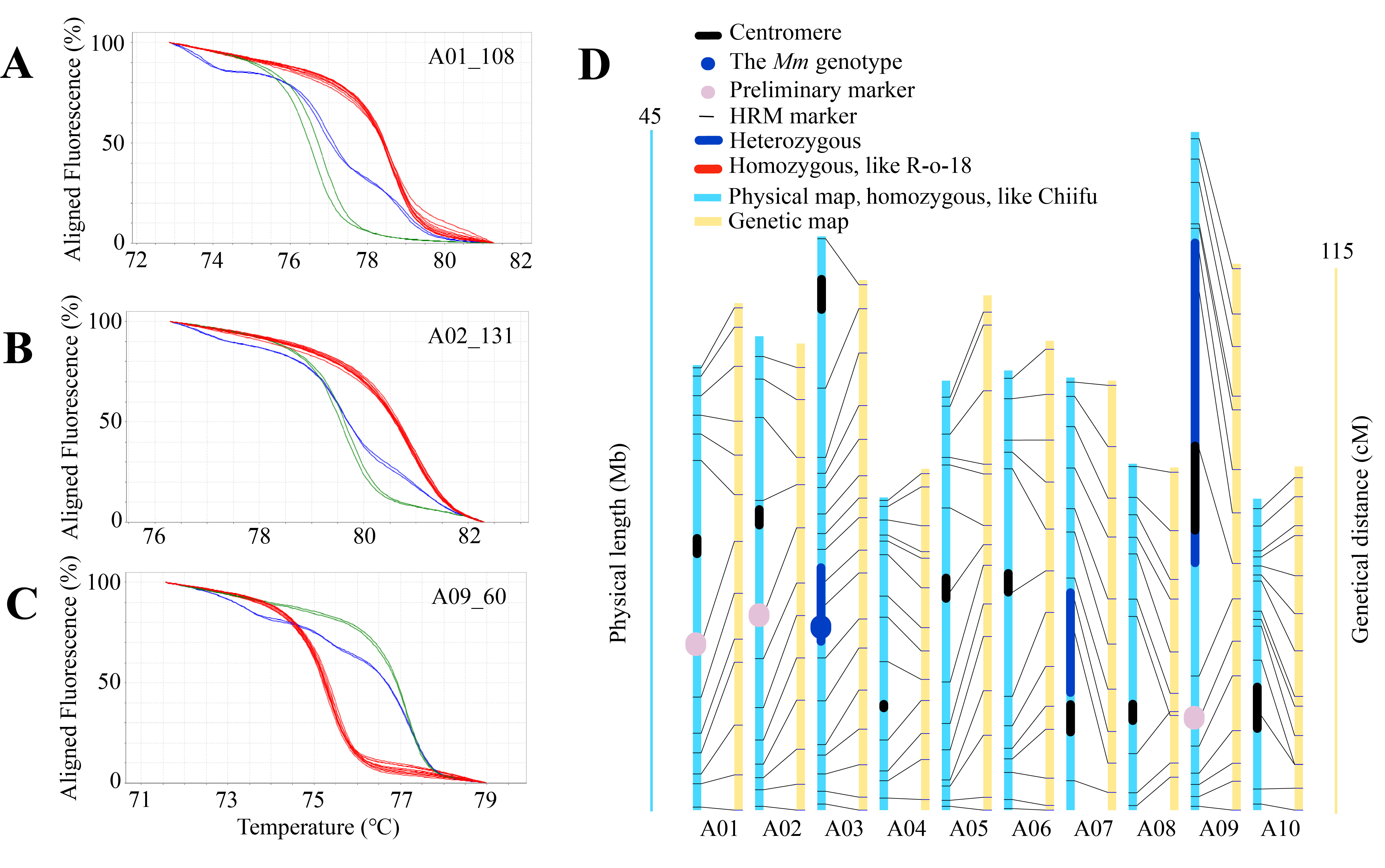


**Supplementary Figure 4.** Genetic background analysis by 131 HRM Markers and WGR. **(A-C)** Genotyping by three co-dominant HRM markers (A01_108, A02_131, A09_60) serving as preliminary markers located on chromosomes A01, A02, and A09, respectively. Red curved lines represent genotypes identical to Chiifu, while green curved lines represent genotypes identical to R-o-18. Blue curved lines indicate heterozygous genotypes. **(D)** Genotypes (genotyping by 131 HRM markers including three preliminary markers) were anchored to the physical map and genetic map in BC_3_-1. Light blue segment indicated a genotype identical to Chiifu, navy blue segment represented a heterozygous genotype. The navy blue dot represents the *Mm* genotype. The pink dots represent the positions of three preliminary markers.


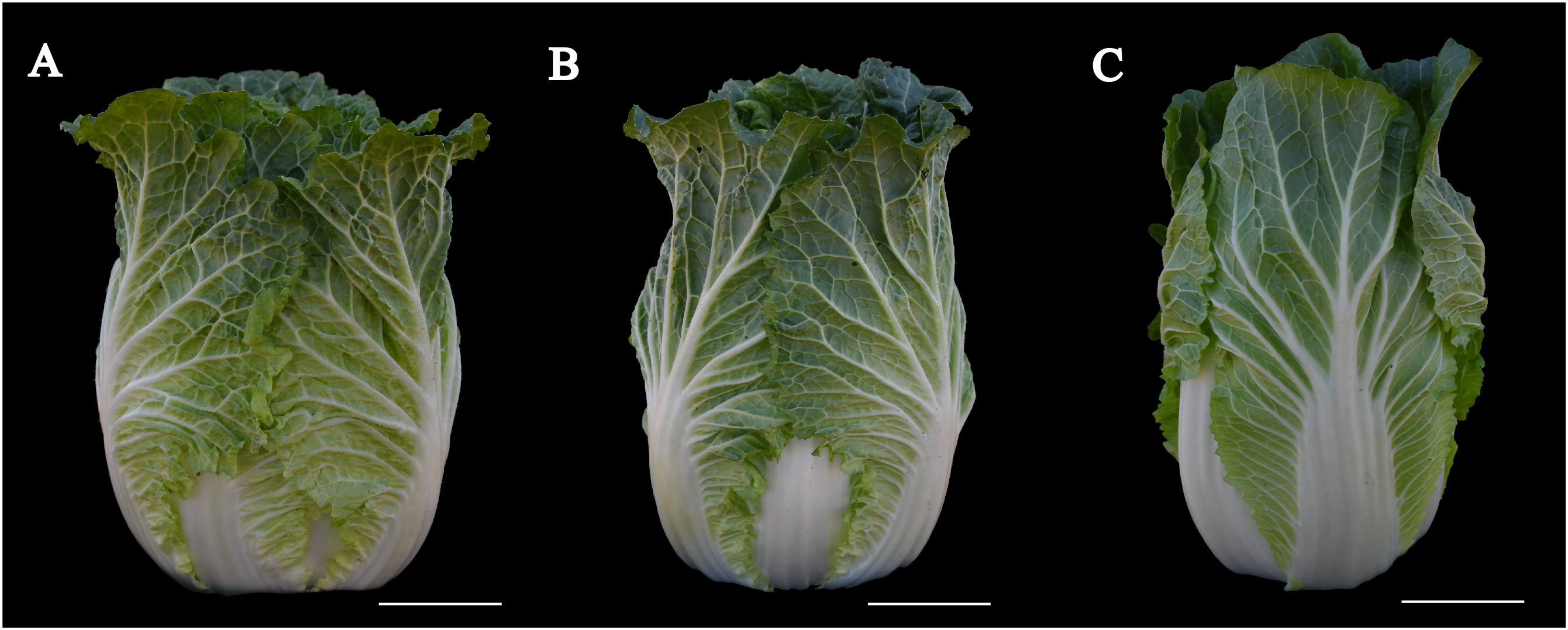


**Supplementary Figure 5.** The phenotypes of Chiifu, BC_3_-1S_1_-6 and BC_3_-7S_1_-14 individual plants. **(A)** The phenotype of Chiifu. **(B)** The phenotype of BC_3_-1S_1_-6 individual plant. **(C)** The phenotype of BC_3_-7S_1_-14 individual plant. Scale bars in (A to C), 5 cm.


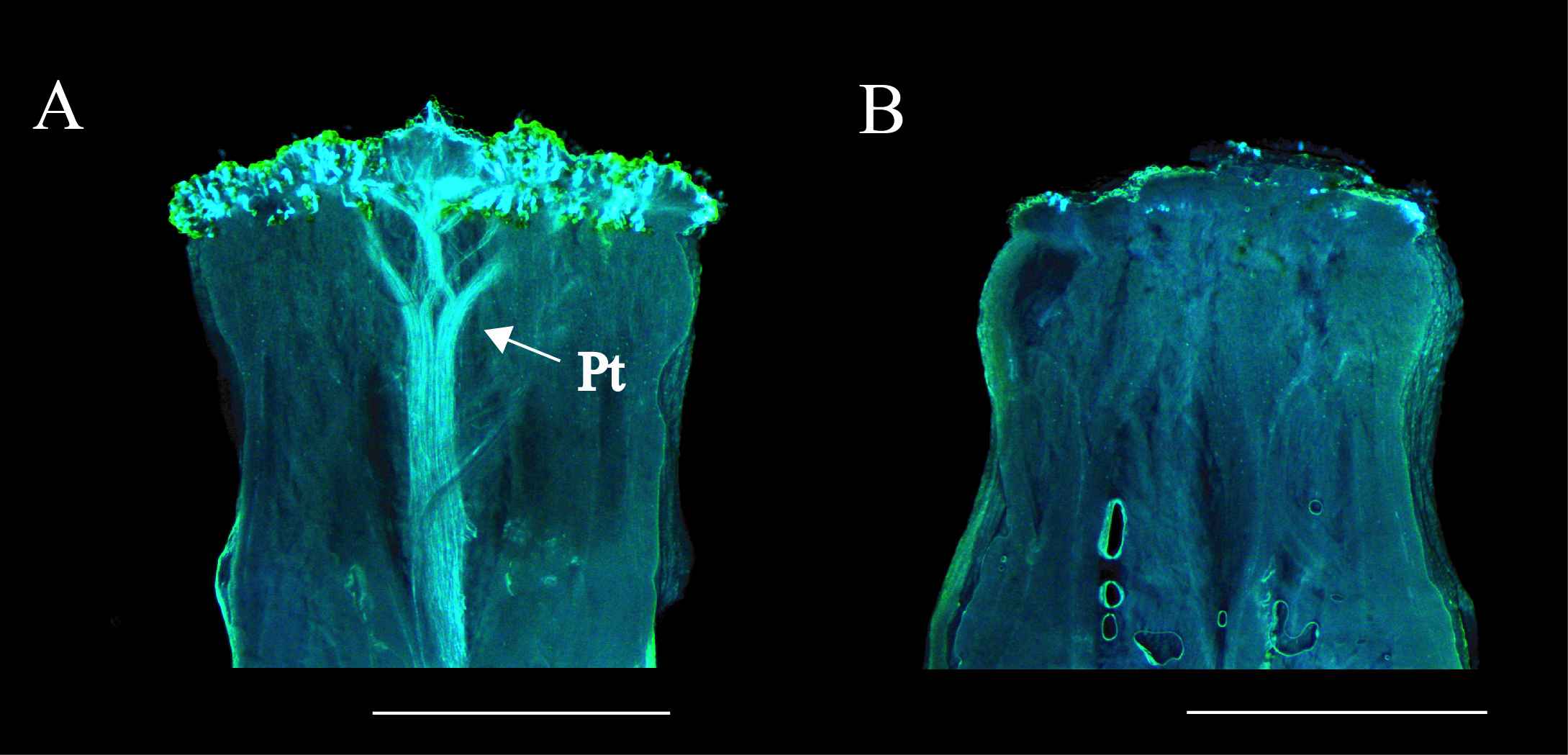


**Supplementary Figure 6.** Fluorescence microscopic images depicting pollen tube germination at the stigmas of BC_3_-7S_1_-14 and BC_3_-1S_1_-6, with “Pt” indicating the pollen tubes. **(A)** BC_3_-7S_1_-14. **(B)** BC_3_-1S_1_-6. Scale bars in (A to B), 1 mm.
